# Practical Departments

**Published:** 1896-09-05

**Authors:** 


### PRACTICAL DEPARTMENTS.

### THE CHEMISTS' EXHIBITION.

A very interesting exhibition of the objects dealt in by chemists has been recently held in London. The exhibition was organised by the *British and Colonial Druggist*, and great credit is due to the management for the arrangement of one of the most complete displays that has ever been got together of the very wide range of products for which people nowadays go to their druggists.

Although intended to appeal more especially to those whose trade it is to deal in drugs, the exhibition contained much that was of interest to physicians and surgeons, showing as it did the cleanly, accurate, and palatable means of drug administration which are placed at their disposal by the resources of modern wholesale pharmacy. Who would like to return to the ordinary pill after examining the beautiful specimens of coated pills shown by many of the firms in this exhibition, among whom we may name Bleasdale, of York; Howard, Lloyd, and Co., of Leicester; Southall Brothers, Parke, Davis, and Co., and others?

But the mere coating of pills is only a detail. Efforts have been made to render them easier to swallow by making them oval, as is done by Wyleys, of Birmingham, or by coating them with sugar, as is done by Southall Brothers and by Parke, Davis, and Co. But pills, however coated, are not the finest product of the art of pharmacy. Almost the whole range of dry drugs is now available compressed into the form of tabloids or tablets, as witness the fine exhibit of Burroughs, Wellcome, and Co., and the samples shown by Hockin, Wilson, and Co.; and when these tabloids are coated with sugar, as in the case of the Bland Pill Tabloids of the former of these firms, the act of swallowing them is still further facilitated.

Messrs. Oppenheimer, however, in their exhibitions of palatinoids and bi-palatinoids show what may be spoken of as a still further refinement, the drugs being enclosed in a capsule, uncompressed and unmixed with any other substance, and where it is desirable that the components of the drug should be kept apart, so that they may only re-act on each other at the moment of being swallowed, such components are kept in separate chambers of the palatinoid until the coating dissolves in the stomach and sets them both free. These are niceties of pharmacy that our fathers would not have dreamed of.

Turning again to the surgical side, any one who remembers the roll of lint, the roll of plaster, and the common calico bandages, which formed the basis of all surgical dressings only a quarter of a century ago, and who turns to the admirable exhibits shown by John Milne, of Ladywell, and of St. Dalmas and Co., of Leicester, will not only be struck with the great variety of dressings used, which is not altogether an unmixed blessing, but with the nicety with which they are now prepared for the surgeon's use, and the facilities which are now at his disposal for obtaining clean, separate, and untouched dressings for each case, and for keeping such dressings unchanged, unsoiled, and in perfect condition for an indefinite time, ready for all emergencies. From a surgical point of view John Milne's dressings, ligatures, &c., and the way in which they are put up, are worthy of all commendation. Again, among the surgical instruments we saw much to admire in the exhibit of Maw, Son, and Thompson.

Naturally, this exhibition included a considerable display of disinfectants, among which we may notice the long list of disinfecting substances shown by the Sanitas Company, who have now extended their trade so as to include the supply of eucalyptus oils and the carbolic and coal tar series of disinfectants, as well as the Sanitas compounds for which they have so long been known.

We have not space here to do more than allude to the large number of food products which are now dealt in by druggists, such as the beef juice shown by Burroughs, Wellcome, and Co., the extract of beef by the Liebig's Extract of Meat Company, and the preserved milk shown by the "Excel" Sterilised Milk Company.

### LAUNDRY AND SANITARY EXHIBITION.

The Exhibition of Laundry and Sanitary Appliances, opened at the Agricultural Hall, Islington, from August 24th to September 5th, is the fourth of its kind, promoted, like its predecessors, by the energy of Messrs. Cordingly and Co. It has been well worthy of a visit from all interested in laundries and their management, giving, as it did, an excellent show of the best and most modern machinery in that important domestic department, and including also appliances connected with baths and wash-houses, disinfecting and sanitary apparatus, and the like. The exhibits of Messrs. Armstrong, Summerscales, and Broadbent were interesting as always, and among them were to be found the newest inventions in laundry machinery. The Blackman Ventilating Company's new air-warmer, applied to a drying-room, especially deserved attention, intended as it is to fix over any existing iron heating stove, and so utilising heat, otherwise wasted, for drying and airing purposes. The same company had on view, in operation, their now well-known "Rapid Drying Room." Water-softeners and purifiers, important adjuncts to town laundries, were numerous, and enamelled and glazed steeping tanks and wash-tubs were shown by Messrs. Cliff and Sons. A special feature of the exhibition this year consisted in prizes offered for the best show of finished laundry work, many of the exhibits being beautifully got up.

